# The Effect of Contrast Enhanced Abdominopelvic Magnetic Resonance Imaging on Expression and Methylation Level of *ATM* and *AKT* Genes 

**DOI:** 10.22074/cellj.2021.7258

**Published:** 2021-07-17

**Authors:** Amir Hossein Jalali, Hossein Mozdarani, Hossein Ghanaati

**Affiliations:** 1 Department of Medical Genetics, Faculty of Medical Sciences, Tarbiat Modares University, Tehran, Iran; 2 Advanced Diagnostic and Interventional Radiology Research Center, Tehran University of Medical Sciences, Tehran, Iran

**Keywords:** 3 Tesla Magnetic Resonance Imaging, Contrast Media, Gene Expression, Methylation Cell Journal(Yakhteh), Vol 23, No 3, August 2021, Pages: 335-340

## Abstract

**Objective:**

To evaluate the effect of contrast enhanced abdominopelvic magnetic resonance imaging (MRI), using a 3 Tesla scanner, on expression and methylation level of *ATM* and *AKT* genes in human peripheral blood lymphocytes.

**Materials and Methods:**

In this prospective *in vivo* study, blood samples were obtained from 20 volunteer patients with mean age of 43 ± 8 years (range 32-68 years) before contrast enhanced MRI, 2 hours and 24 hours after contrast enhanced abdominopelvic 3 Tesla MRI. After separation of mononuclear cells from peripheral blood, using Ficoll-Hypaque, we analyzed gene expression changes of *ATM* and *AKT* genes 2 hours and 24 hours after MRI using quantitative reverse transcription polymerase chain reaction (qRT-PCR). We also evaluated methylation percentage of the above mentioned genes in before, 2 hours and 24 hours after MRI, using MethySYBR method.

**Results:**

Fold change analysis, in comparison with the baseline, respectively showed 1.1 ± 0.7 and 0.8 ± 0.5 mean of gene expressions in 2 and 24 hours after MRI for *ATM*, while the results were 1.4 ± 0.6 and 1.4 ± 1 for *AKT* (P>0.05). Methylation of the *ATM* gene promoter were 8.8 ± 1.5%, 9 ± 0.6% and 9 ± 0.8% in before contrast enhanced MRI, 2 and 24 hours after contrast enhanced MRI, respectively (P>0.05). Methylation of *AKT* gene promoter in before contrast enhanced MRI, 2 hours and 24 hours after contrast enhanced MRI was 5.4 ± 2.5, 5 ± 3.2, 4.9 ± 2.9 respectively (P>0.05).

**Conclusion:**

Contrast enhanced abdominopelvic MRI using 3 Tesla scanner apparently has no negative effect on the expression and promoter methylation level of *ATM* and *AKT* genes involved in the repair pathways of genome.

## Introduction

Magnetic resonance imaging (MRI) is a powerful and relatively safe diagnostic imaging modality, commonly used to visualize internal organs of the human body. In comparison with computed tomography (CT) scan, using static and gradient field combined with radiofrequency (RF), MRI provides higher contrast among the different body tissues such as brain, abdominopelvic and cardiovascular system (1).

Although it is proved that ionizing radiation, such as X-rays or γ-radiation, may cause DNA damage, there are unresolved questions about health risks due to non-ionizing radiation (2). The increased exposure to non-ionizing radiation from wireless communication devices, power lines and MRI caused new safety concerns (3).

Due to the high number of MRI scans performed in the world and the usage of high-field machines operating at high magnet field levels, any evidence of possible genotoxic effects of MRI needs meticulous consideration.

There are contradictory results about the genetic damage of MRI on human blood cells of individuals exposed to different fields of MRI. While some articles mentioned enhanced DNA damage in human lymphocytes after MRI (4-8), others did not approve these findings (1, 9-16). Besides, radiocontrast agents which are frequently used in diagnostic radiology as well as MRI may cause genotoxicity (17-19). In the studies reporting DNA damage after MRI, the most important finding is DNA-double strand break (DNA-DSB). Knowledge is now incomplete about cytotoxicity due to the complex way of response to genotoxins by evoking cellular processes that may finally lead to DNA repair, damage fixation as mutations or damage removal by different routes of cell death (20, 21). 

Many studies showed gene up-regulations involved in signal transduction process, cell cycle, DNA repair and apoptosis after radiation exposure in different cells (22, 23).

It seems that **AKT** activation is an important event in the induction of radiocontrast agent mediating side effects and inhibition of *AKT* activity impairs repair of DNA-DSB (24). As a large number of MRI examinations reperformed by contrast media and due to the effect of some contrast agents on AKT expression we have considered this repair gene to evaluate the safety of contrast enhanced MRI.

Besides, Ataxia telangiectasia mutated (*ATM*) gene encodes a serine threonine protein kinase activated by sensing DNA-DSB (25).

DSB induced by irradiation, leads to activation and phosphorylation of ATM, cell-cycle checkpoints and DNA repair proteins. Besides, X-irradiation can induce up-regulation of *ATM* gene expression in lymphoblastoid cell lines (26). Halm et al. (27) found that CT scan exposure can alter *ATM* gene expression. One important note about tumor suppressor genes is that they can be inactivated by their promoter methylation and many environmental factors can change DNA methylation patterns of human cells (25).

To the best of our knowledge, there are limited studies about the effect of ionizing radiation on gene expression and DNA methylation. In addition, there is no study about the effect of MRI on gene expression and methylation. 

In this study, we aimed to assess the effect of contrast enhanced abdominopelvic MRI using a 3 Tesla scanner on expression and methylation level of *ATM* and *AKT* genes in human peripheral blood lymphocytes.

## Materials and Methods

Written informed consent was obtained from all patients. The study was performed in accordance with the Declaration of Helsinki and approved by Ethics Committee of Tarbiat Modares University (Tehran, Iran, IR.TMU.REC.1396.585). Patients with a history of malignancy, inflammatory or autoimmune diseases, receiving any chemo- or radio-therapy, being smoker and performed medical imaging during the last three months were excluded from the study.

In this prospective *in vivo* study, twenty volunteer patients (15 women and 5 men) referred for abdominopelvic MRI to the imaging center, contributed to this study. The mean age of our studied cases was 43 ± 8 years (range: 32-68 years). The mean body weight of our patients was 66.5 ± 13.5 kilogram (range: 45-90) and their mean height was 162.4 ± 6.6 centimeter (range: 150-175). Final diagnosis of our patients was uterine fibroids in five, ovarian simple cyst in three and liver hemangioma in three cases while nine cases were normal. Sample size was calculated for comparison of two means, considering that type I and II statistical errors were 0.05 and 0.2. All parameters of the formula were extracted from the study performed by Lee et al. (5).

Contrast enhanced abdominopelvic MRI was performed by 3 Tesla MRI machine (Discovery, USA) equipped with a maximum gradient strength amplitude per axis of 50 mT/m and a maximum slew rate per axis of 200 T/m/sec. Pelvic MRI standard sequences were sagittal and coronal T2 fast spin-echo (FSE), axial T2, T2 fat suppression and T1 FSE, axial multi b-value diffusion-weighted imaging (DWI) 50, 400 and 800 seconds/mm^2 ^, coronal, sagittal and axial T1 FSE FS post contrast injection. The abdomen MRI protocol included coronal and axial T2 single-shot FSE (SSFSE), axial fast imaging employing steady-state acquisition (FIESTA) and 3D T1 GE FS liver acquisition with volume acceleration (LAVA), axial multi b-value DWI 50, 500 and 1000 seconds/mm^2^ , coronal and axial post-IV GBCA 3D T1 LAVA FS sequences. Gadoterate meglumine (Dotarem, Guerbet, France, 0.2 mL/kg, 0.1 mmol/kg) was administrated using injector. Using antecubital vein, 5 ml of peripheral blood were drawn from each patient before MRI, 2 hours and 24 hours after MRI. 

Blood samples were collected in ethylenediaminetetraacetic acid (EDTA) for the separation of mononuclear cells from whole blood using Ficoll-Hypaque (Lymphodex, Germany).

### Evaluating expression of *ATM* and *AKT* genes 

To analyze mRNA expression, we extracted RNA from peripheral blood mononuclear cells (PBMCs) using a total RNA extraction kit (Yekta Tajhiz Azma, Iran) based on the manufacturer’s protocol. We quantified concentration of RNA using a NanoDrop (IMPLEN, Germany) and the purity of RNA was evaluated by the 260/280 nanometer absorbance ratio. After RNA extraction, complementary DNA (cDNA) was synthesized by using a synthesis kit based on the manufacturer’s protocol. Human β-Actin (*ACTB*) gene was applied as internal control to normalize input RNA amount, reverse transcription efficiency and RNA quality.

mRNA levels of target genes, including *ATM*, *AKT*, as well as housekeeping gene (*ACTB*) were measured by semi-quantitative reverse transcription polymerase chain reaction (PCR) using SYBR Green detection kit (Biofact, South Korea). Primers of the targeted genes are shown in Table 1.

**Table 1:** Primer sequences of the target genes to evaluate gene expression


Gene	Primer sequence (5ˊ-3ˊ)	Size (bp)	TM (˚C)

*ATM*	F: GCCTGATTCGAGATCCTGAAAC	22	62.1
	R: GGCTTGTGTTGAGGCTGATAC	21	61.3
*AKT*	F: AAGAAGCTCCTGCCACCCTT	20	60.5
	R: CAGTAAGCCCAGGCTGTCATAG	22	64
*BETA ACTIN*	F: TGGATGATGATATCGCCG	18	53.9
	R: CACGATGGAGGGGAAGAC	18	58.4


TM; Melting temperature.

Duplicate repeat was performed for each sample in a StepOnePlus™ Real-Time PCR System (Applied Biosystems, USA). The temperatures set was one cycle of 95˚C (pre-denaturation) for 10 minutes followed by 40 cycles including 15 seconds of denaturation at 95˚C, 30 seconds of annealing at 60˚C, and 10 seconds of extension at 72˚C.

LinReg software was used for calculation of the PCR efficiency and the relative expression of genes was measured according to the method previously reported by Pfaffl and his colleagues (28). 

### Evaluating methylation of *ATM* and *AKT* genes promoter

Genomic DNA was isolated from PBMCs using a DNA extraction Kit (Yekta Tajhiz Azma, Iran) based on the manufacturer’s protocol. We assessed the quality of DNA by utilizing an absorbance ratio of 260 nm to 280 nm (A_260/A280_) by a NanoDrop. We considered the samples with the absorbance ratio of 1.8-2.0 as good quality. Sodium bisulfite treatment of genomic DNA was done using the protocol described by Herman et al. (29) with modifications, as reported previously. Sodium bisulfite treatment changes unmethylated cytosine to uracil, whereas methylated cytosines exist unchanged. After bisulfite treatment, we aliquoted DNA samples at 80˚C. In this study, we used one-step MethySYBR method to calculate methylation quantitatively. By this method, bisulfite modified DNA was amplified in two concurrent real-time PCR reaction. The primers applied for MethySYBR are presented in Table 2.

**Table 2:** Primer sequences to evaluate methylation in the target genes


Primer	Primer sequence (5ˊ-3ˊ)	Size (bp)	TM (˚C)

*ATM-Methylated*	F: GTTTTGGAGTTTGAGTTGAAGGGT	24	55.8
	R: AACTACCTACTCCCACTTCCAA	22	55.1
*ATM-Outer*	F: GAGGGTGGGTGAGAGTTT	18	50.9
	R: CCCCTACCACTACACTC	17	54
*AKT-Methylated*	F: GGGTGTTTTTGCGGGTCG	18	57.5
	R: CGACCGCGACGAATCTTTC	19	56.4
*AKT-Outer*	F: GGTTTGGAGTTGGGGTT	17	52.4
	R: AAACCCTCCCACAAACTTAAAAAC	24	54.2


TM; Melting temperature.

In the first reaction, DNA was amplified, regardless of the methylation status and it was used as reference control for normalization of the methylated alleles in the second reaction. In this method, fully methylated DNA is used as a calibrator to measure the methylation percentage.

PCR conditions for *ATM* methylation were 95˚C for 10 minutes, followed by 40 cycles of 95˚C for 15 seconds, 58˚C for 30 seconds and 72˚C for 10 seconds. PCR conditions for *AKT* methylation were 95˚C for 10 minutes, thereafter followed by 40 cycles of 95˚C for 15 seconds, 57˚C for 30 seconds and 72˚C for 10 seconds.

The cycle threshold (C_t_) value of amplified DNA was retrieved from the C_t_ of amplified methylated DNA to acquire the sample’s and calibrator’s ΔC_t_ values. For calculation of methylation percent of each sample, fully methylated ΔC_t_ was retrieved from the sample ΔC_t_ to acquire ΔΔC_t_ value, which is then applied into the 2^(−ΔΔCt)^ formula, and multiplied by 100 to show the methylation percentage of samples.

### Statistical analysis

Statistical analyses were performed by SPSS version 16 (SPSS Inc., USA). For normal distributed variables, we used parametric tests (repeated measure ANOVA or paired t test) for comparison of the groups. If variables did not show normal distribution or if data were ordinal, we would use non-parametric tests. We considered P<0.05 as statistically significant.

## Results

### Results of gene expression

**Fig.1:**
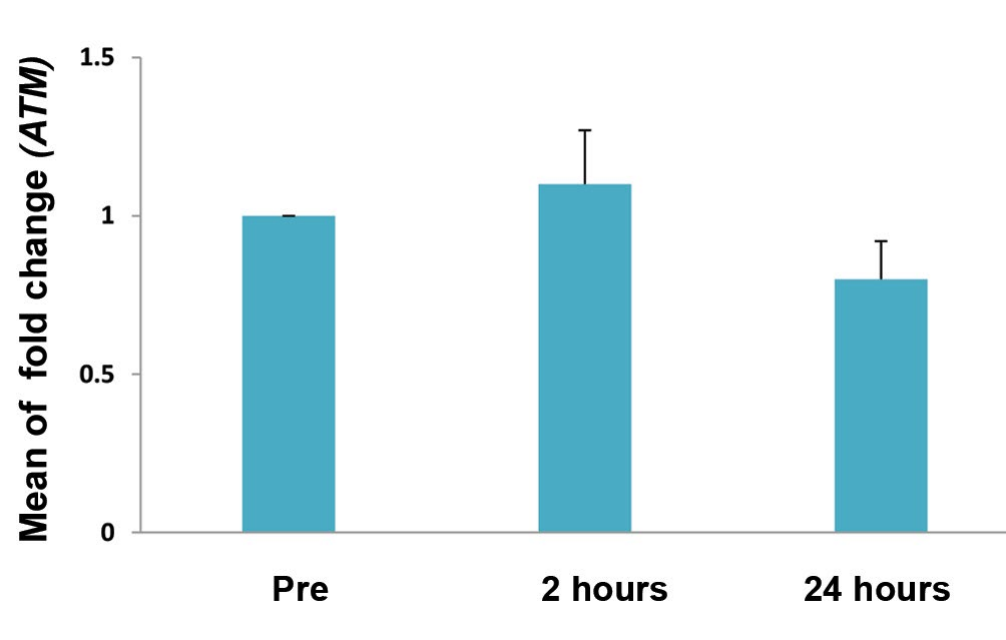
Relative expression (fold change) of mRNA transcripts for *ATM* gene in 20 cases before (Pre), 2 and 24 hours after magnetic resonance imaging (MRI). Error bars represent standard error (SE).

**Fig.2:**
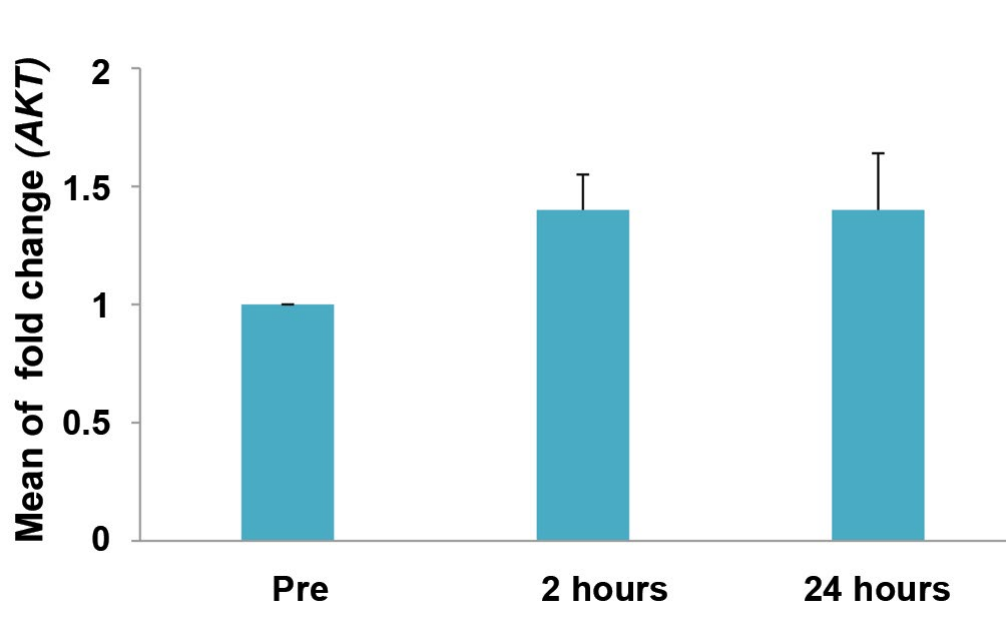
Relative expression (fold change) of mRNA transcripts for *AKT* gene in 20 cases before (Pre), 2 and 24 hours after magnetic resonance imaging (MRI). Error bars represent standard error (SE).

No statistically significant change was seen in expression of *ATM* and *AKT* genes of the cases after contrast enhanced MRI. Mean of gene expressions were 1.1 ± 0.7 and 0.8 ± 0.5 fold change in 2 and 24 hours after contrast enhanced MRI for *ATM* gene (P>0.05, based on paired t test, Fig.1). The results for *AKT* showed that the mean of gene expressions were 1.4 ± 0.6 and 1.4 ± 1 fold change in 2 and 24 hours after contrast enhanced MRI (P>0.05, based on paired t test, Fig.2).

### Results of methylation

**Fig.3:**
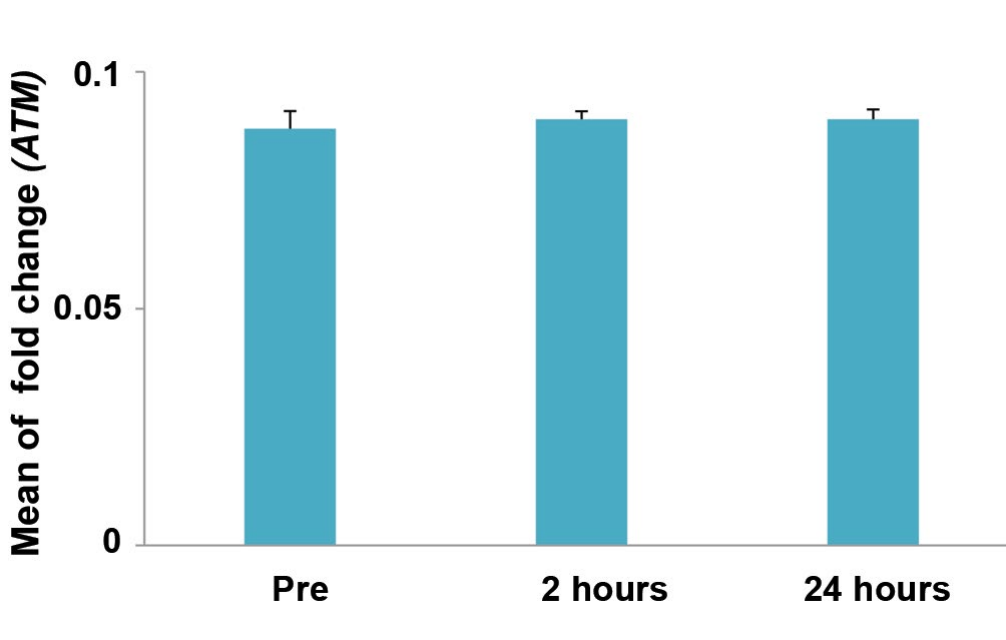
Methylation level of *ATM* gene in 20 cases before, 2 hours and 24 hours after contrast enhanced magnetic resonance imaging (MRI). Error bars represent standard error (SE).

There was not statistically significant change in the methylation percent of *ATM* gene after contrast enhanced MRI. Methylation percent of the *ATM* gene promoter were 8.8 ± 1.5%, 9 ± 0.6% and 9 ± 0.8% in respectively before contrast enhanced MRI, 2 hours and 24 hours after contrast enhanced MRI (P>0.05, based on repeated measure ANOVA, Fig.3).

**Fig.4:**
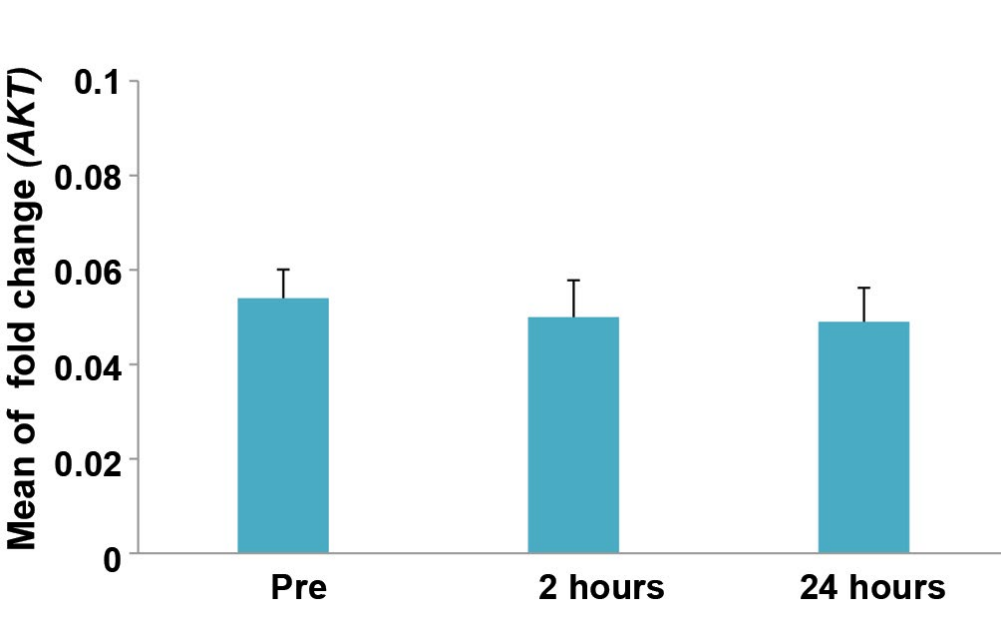
Methylation level of *AKT* gene in 20 cases before, 2 hours and 24 hours after contrast enhanced magnetic resonance imaging (MRI). Error bars represent standard error (SE).

Methylation percent of *AKT* gene promoter in before, 2 hours and 24 hours after contrast enhanced MRI was respectively 5.4 ± 2.5, 5 ± 3.2, 4.9 ± 2.9 showing no statistically significant change in DNA methylation (P>0.05, based on repeated measure ANOVA, Fig.4).

## Discussion

MRI is a non-invasive diagnostic modality in comparison with the other imaging scanners, such as X-ray or CT scan, which have ionizing radiation hazards. However, there are some concerns about the possible MRI risks in recent years which have not been clarified yet.

Despite the ionizing radiation causes DNA damage even at low dosages, energy levels of electromagnetic fields (EMF) applied in MRI are not enough for direct breakage of chemical bonds (30). Besides, we cannot exclude the indirect harmful effects of EMF on DNA integrity. Creation of oxidative stress during MRI might be one possible cause of DNA damage (30, 31). 

After careful search, we found that there are only14 research articles about genotoxic effects of MRI in the literature. The above mentioned studies have a lot of diversity in field strengths (1.5-7 Tesla), exposure factors and genotoxicity evaluation methods. Besides, there is no confirmed hypothesis to explain the possible mechanisms of the molecules significantly affecting this event. Among these reports, five articles mentioned an increase in DSB detected by ƔH2AX, enhanced number of micronuclei or increase of comet formation with alkaline single-cell gel electrophoresis (4-8). In contrast, nine studies did not detect any genotoxic effects after MRI using 1.5-7 Tesla machines (1, 9-16).

In our prospective *in vivo* study, we investigated 3 Tesla MRI and the applied MRI sequences were taken from contrast enhanced abdominopelvic protocols used in our routine clinical examinations. To the best of our knowledge, there is no other study in the literature about the evaluation of possible epigenetic changes after abdominopelvic MRI. Our results indicated that MRI has not adverse effects on the gene expression and methylation of *AKT* and *ATM* genes.

Similar to our study, Brand et al. (1) used Dotarem for contrast enhanced cardiac MRI using 1.5 Tesla scanner and they did not find immediate increase in DNA damage of human lymphocytes. A different contrast media (Gadobutrol) was used in Fiechter et al. (7) study for MRI on 1.5 Tesla scanner by using ƔH2AX immunofluorescence microscopy and they showed a significant increase of DSB. Reddig et al. (12) also used Gadobutrol for evaluation of H2AX foci formation in patients underwent MRI. They found no evidence of DNA damage after MRI with different magnetic fields (1-7 Tesla).

In the other study performed by Yildiz et al. (6), the authors reported that contrast enhanced MRI, using Omniscan, was associated with an immediate increase in single-strand DNA breakage. Although studies reported that DNA damage may occur in peripheral blood lymphocytes during MRI, the concern was expressed since only a single marker was evaluated and downstream consequences have not been evaluated.

All of the mentioned articles have examined the cytotoxic effects of MRI. The only study evaluating the effects of MRI on DNA repair genes has been performed by McDonald et al. (32), in which the authors found a small significant increase in the DNA repair protein 53BP1 after MRI.

Considering that DNA damage factors engage repair proteins, such as *ATM* or DNA-PK (32), evaluation of changes in downstream DNA repair factors might be considered as additional markers for the evaluation of the effects of MRI on DNA.

*ATM* gene produces a protein kinase playing important role in triggering proper cellular response to DNA damage (33) and similar to the other tumor suppressor genes, promoter methylation is the main epigenetic mechanism which can prevent *ATM* transcription (25).

Previous studies showed *ATM* expression changes 1 hour after CT scan from very low radiation dosages, as low as 0.1 Gy (27).

Owing to the results of one study suggesting that extremely low-frequency EFM (ELF-EMF) exposure can induce modification in methylation and expression of DNMTs, epigenetic may have vital role in the biological effects of magnetic exposure (34).

Indeed, *AKT* gene has fundamental role in the cytotoxicity effect of radiocontrast media (RCM) (35).

RCM can influence intracellular signaling pathways and can affect PI3K/Akt pathway via suppressing *AKT* phosphorylation and downstream targets (35, 36). Our study has some limitations need to be mentioned. Firstly, only one contrast media (Dotarem) was studied in our research and we should examine the other contrast agents of MRI. Secondly, we examined only two genes. Using microarray and whole genome methylation assessments, other complementary studies composed of panels of whole genes involved in repair and apoptosis are recommended.

## Conclusion

Contrast enhanced abdominopelvic MRI using 3 Tesla scanner has apparently no negative effect on the expression and promoter methylation levels of two genes involved in the repair pathways of the genome, namely *ATM* and *AKT*. Finally, our results should be interpreted cautiously, since it might not indicate exact evidence whether MRI is safe and it has no adverse effect on DNA. Complementary studies, including evaluation of the other DNA damage and repair markers as well as whole genome methylation, are necessary to understand the MRI safety.
